# The Heterogeneity of Immune Cell Infiltration Landscape and Its Immunotherapeutic Implications in Hepatocellular Carcinoma

**DOI:** 10.3389/fimmu.2022.861525

**Published:** 2022-03-10

**Authors:** Yuanyuan Guo, Jing Yang, Kaidi Ren, Xueke Tian, Hua Gao, Xin Tian, Xiaojian Zhang, Quancheng Kan

**Affiliations:** ^1^ Department of Pharmacy, The First Affiliated Hospital of Zhengzhou University, Zhengzhou, China; ^2^ Henan Key Laboratory of Precision Clinical Pharmacy, Zhengzhou University, Zhengzhou, China; ^3^ Department of Radiotherapy, The First Affiliated Hospital of Zhengzhou University, Zhengzhou, China

**Keywords:** immune cell infiltration, tumor heterogeneity, immune microenvironment, hepatocellular carcinoma, tumor mutational burden

## Abstract

Immunotherapy, closely associated with immune infiltration and tumor mutation burden (TMB), is emerging as a promising strategy for treating tumors, but its low response rate in hepatocellular carcinoma (HCC) remains a major challenge. Herein, we applied two algorithms to uncover the immune infiltration landscape of the immune microenvironment in 491 HCC patients. Three immune infiltration patterns were defined using the CIBERSORT method, and the immune cell infiltration (ICI) scores were established using principal component analysis. In the high ICI score group, the activation of the Wnt/β-catenin pathway was significantly enriched and expressions of immune checkpoint genes increased, which showed a pessimistic outcome. The low ICI score group was characterized by increased TMB and enrichment of metabolism-related pathways. Further analysis found that the ICI score exhibited a significant difference in age ≥65/age <65, grade I/grade II–IV, and response to immunotherapy. Moreover, the CTNNB1 mutation status was found to be closely associated with prognosis and immunotherapeutic efficiency, significantly affecting the ICI score and TMB, which might be regarded as a potential marker for the treatment of HCC. The evaluation of immune infiltration patterns can improve the understanding of the tumor immune microenvironment and provide new directions for the study of individualized immunotherapy strategies for HCC.

## Introduction

Liver cancer ranks as the fourth leading cause of most common malignancies in the world (1, 2), 90% of which is hepatocellular carcinoma (HCC). At present, the main treatment options are liver transplantation, transarterial chemoembolization, radiofrequency ablation, surgery in the early stage, and molecular targeting agents (e.g., sorafenib, regorafenib, and lenvatinib) in the advanced stage (3). Although these treatment strategies lead to a modest survival benefit, the overall survival of HCC is still challenging due to the heterogeneity of HCC.

Immunotherapy can recognize and eliminate tumor cells by activating and enhancing the host immune system. Emerging evidence has highlighted that immunotherapy has been clinically proven to be an effective treatment for a variety of cancers (4). However, a major limitation is that only 10% to 20% of cancer patients can benefit from this treatment, which could be due to the difference in the amount of immune cell infiltration or somatic variants in tumor types (5). Therefore, it is an urgent need to identify the new therapeutic markers to determine the ideal HCC subgroups for immunotherapy.

Immune cells and stromal cells constitute the main part of the tumor microenvironment (TME), which are responsible for tumor spread, recurrence, metastasis, the effect of immunotherapy, and prognosis (6–9). For example, tumor-infiltrating CD4^+^ and CD8^+^ T lymphocytes play an antitumor role, which is associated with favorable prognosis (10–14). Tumor-associated macrophages (TAMs) exert a tumor-promoting effect by secreting immunosuppressive factors, thereby reducing survival outcomes (15, 16). In addition, excessive invasion of stromal components in tumor tissues hinders the transport of immune cells to tumor, suggesting that the intercellular communication in TME, rather than the single-cell population, is more likely to affect the occurrence and development of tumors (17–20). Thus, it is more important to elucidate the composition and characteristics of the complex tumor immune medium than in single-cell populations in TME.

In the study, we performed two calculation algorithms, Cell-type Identification by Estimating Relative Subsets of RNA Transcripts (CIBERSORT) and ESTIMATE, to analyze the gene-expression profiles of HCC samples and comprehensively describe the immune landscape of HCC. Besides, we classified HCC samples into three immune subtypes according to the immune infiltration patterns. The ICI score was established to characterize various immune statuses, which can accurately predict HCC patients’ response and prognosis to immunotherapy.

## Materials and Methods

### Data Collection From TCGA and GEO Databases

A total of 376 HCC samples were downloaded from TCGA database (https://portal.gdc.cancer.gov/), and 115 samples were downloaded from GSE76427 (https://www.ncbi.nlm.nih.gov/geo/query/acc.cgi?acc=GSE76427). The detailed description of the clinical information of HCC samples is provided in **Supplementary Table S1**. Another 80 samples from GSE10141 were used as an external validation set. To be consistent with the data of the microarrays (21), the fragments per kilobase million (FPKM) values in TCGA dataset were normalized as TPMs (transcripts per kilobase million). Then the TCGA-LIHC and GEO data were converted to log2 (TPM +1) to avoid negative values. Meanwhile, the deviation between different datasets caused by batch effects was removed using the “ComBat” algorithm (22).

### Immune Profile Analysis

The “CIBERSORT” (Cell-type Identification by Estimating Relative Subsets of RNA Transcripts) algorithm was used to assess the proportion of distinct immune cells in each sample from TCGA-LIHC and GSE76427 datasets. The principle was to infer the proportion of cell types from tumor samples with mixed cell types using support vector regression based on the signature of 22 different immune cells (LM22, including B cells, T cells, natural killer cells, monocytes, macrophages, dendritic cells, and mast cells). Using the gene expression signature of LM22 as a reference, the “CIBERSORT” R package was used to infer the composition of immune cells in HCC samples by the deconvolution algorithm (23).

The “MCP-counter” (24) (Microenvironment Cell Populations-counter) algorithm was used to obtain information about the abundance of distinct specific cell lineage (including nine different cell types such as T cells, cytotoxic T cells, NK cells, and monocytic lineage) infiltration in HCC samples with or without CTNNB1. It was also a deconvolution method that used marker genes to quantify the relative abundance of immune cells by the “MCPcounter” R package.

### Tumor Microenvironment Analysis

ImmuneScore, StromalScore, and ESTIMATEScore were quantified for each HCC sample by the ESTIMATE algorithm (25). ImmuneScore captured the infiltration of immune cells, while StromalScore represented the presence of stroma in tumor tissue. Specifically, ESTIMATEScore was calculated as the sum of ImmuneScore and StromalScore.

### Consensus Cluster of Tumor-Infiltrating Immune Cell Analysis

According to the immune cell infiltration profiles, the number of unsupervised clusters was assessed *via* the “ConsensuClusterPlus” R package (26). The unsupervised clustering “Pan” method was performed based on Euclidean and Ward’s linkage analysis, and the procedure was repeated 1,000 times to ensure the classification stability. The result was visualized using the “heatmap” R package, and the subtype assignments were verified using t-distributed stochastic neighbor embedding (t-SNE).

### Differential Gene Screening of Different Immune Infiltration Patterns

The molecular characteristics of different immunophenotypes were further explored by screening differential genes. *p* < 0.05 and absolute fold change >1.5 were set as the cutoff criteria, which was implemented using the “Limma” R package after running a log2 (TPM + 1) transformation.

### Immune Cell Infiltration Score (ICI Score) Algorithm

According to gene expression, the unsupervised clustering method was used to classify HCC patients. Genes positively correlated with clusters were named gene signatures A, and genes negatively correlated were named gene signatures B. The dimension reduction of gene signatures A and B was performed by the “Boruta” algorithm. The signature score was extracted using principal component analysis (PCA). Then, the ICI score for each sample was defined using a method similar to the Gene expression Grade Index (27) as follows:


Immune cell infiltration (ICI) score=∑ PC1A−∑ PC1B

In the formula, PC1A and PC1B represented the first component of gene signature A and gene signature B, respectively.

### Collection and Assessment of Tumor Mutation Burden

The somatic alteration data of the TCGA-LIHC cohort were downloaded from the TCGA data portal website (https://www.cancer.gov/tcga/). The tumor mutation burden (TMB) for each sample was determined by counting the total number of variants through the full length of exons. The “Maftool” R package (28) was used to identify the oncogenic drivers of HCC, and the somatic alterations of the oncogenic drivers were compared between high and low ICI scores. The top 20 oncogenic drivers with the highest alteration frequency were further analyzed to screen potential targets for immunotherapy.

### Gene Set Enrichment Analysis

Gene set enrichment analysis (GSEA) was performed to compare the differences of a predefined set of genes between the high and low ICI score groups. The “c2.cp.kegg.v7.4symbols” downloaded from MSigDB v7.4 (Molecular Signatures Database) was chosen as the reference to calculate the gene set enrichment scores using GSEA software (v4.1.0). NOM *p*-value < 0.05 and FDR *q*-value <0.05 were considered as significant.

### Evaluation of the Immunotherapy Efficacy

The response to immunotherapy was predicted by the Immune Cell Abundance Identifier (http://bioinfo.life.hust.edu.cn/ImmuCellAI) website. The difference of the ICI score between response and non-response groups was analyzed by the Wilcoxon test. Besides, a total of 127 HCC cases with mutation information were downloaded from the cBioPortal database (https://www.cbioportal.org/). Patients who received immunotherapy were analyzed to verify the effect of CTNNB1 mutation on immunotherapy.

### Statistical Analyses

All statistical analyses were performed using SPSS version 22.0 or GraphPad Prism version 7.0 or R version 4.0.3 software. The Kaplan–Meier curve was employed for survival curve analysis with the log-rank test. Two groups were compared by the Wilcoxon test, and three or more groups were compared by the Kruskal–Wallis test. Correlation analysis was performed using the Spearman rank test. A *p* value less than 0.05 was regarded as statistically significant.

## Results

### ImmuneScore Revealed More Prognostic Value in HCC Versus StromalScore

**Figure 1:**
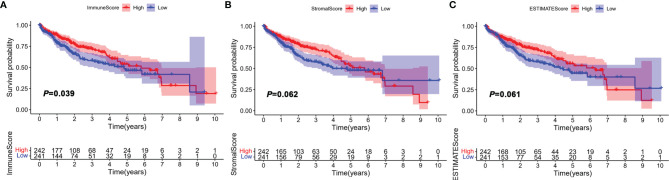
Correlation between ImmuneScore, StromalScore, and ESTIMATEScore with overall survival (OS). HCC patients were divided into two groups according to the median of ImmuneScore, StromalScore, and ESTIMATEScore. High group, n = 242; low group, n = 241. **(A)** Kaplan–Meier curve analysis of ImmuneScore with the log-rank test; **(B)** Kaplan–Meier curve analysis of StromalScore with the log-rank test; **(C)** Kaplan–Meier curve analysis of ESTIMATEScore with the log-rank test.

The workflow of the study design is illustrated in **Figure S1**. In this study, a total of 491 HCC samples from the TCGA-LIHC and GSE76427 were included. The average age of these patients was 60.4 years, and 70.7% of the patients were male, which was 2.41 times higher than that of women (**Supplementary Table S1**). To assess the infiltrating proportion of immune/stromal cells, the ImmuneScore, StromalScore, and ESTIMATEScore for each sample were calculated by the ESTIMATE algorithm, respectively. ImmuneScore ranged from -931.61 to 3,171.87, while the distribution of StromalScore was -1,632.97 to 1,212.68 (**Supplementary Table S2**). The median of ESTIMATEScore was -186.74 ranging from -2,564.58 to 3,808.98, which was calculated by integrating the two scores. According to the median, HCC patients were divided into high and low groups, and the survival analysis was performed to estimate the prognostic value of the three scores. The log-rank test showed that the difference in immune score was statistically significant, and HCC patients with low scores had poor survival outcomes (**Figure 1A**, *p* = 0.039). However, StromalScore and ESTIMATEScore showed no significant correlation with the overall survival (**Figures 1B, C**, *p* = 0.062, *p* = 0.061, respectively). These results suggest that the immune status of TME was more suitable for predicting the prognosis of HCC patients.

### The Landscape of Immune Cell Infiltration in the TME of HCC

**Figure 2:**
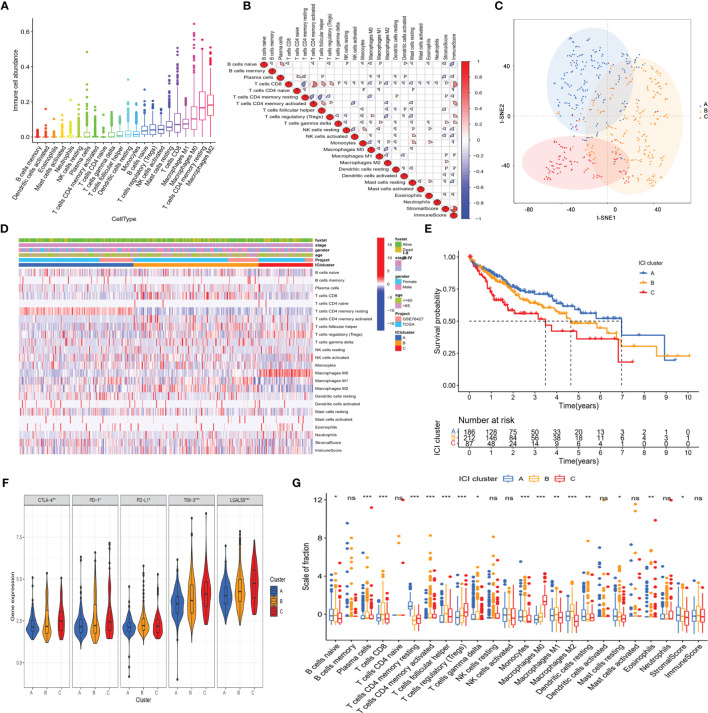
The landscape of immuno-cell infiltration in the TME of HCC. **(A)** Comparison of the abundance of 22 tumor-infiltrating immune cells. **(B)** Correlation between the abundance of 22 tumor-infiltrating immune cells and Immune scores as well as Stromal scores. **(C)** Confirmation of the clustering patterns using the t-SNE algorithm. **(D)** Unsupervised clustering for all HCC patients based on the proportion of immune cells. **(E)** Kaplan–Meier survival analysis of three immune infiltration clusters. **(F)** Comparison of the expression of immune checkpoints among distinct immune infiltration clusters. ^*^ *p* < 0.05; ^**^ *p* < 0.01; ^***^ *p* < 0.001. **(G)** Comparison of fraction of tumor-infiltrating immune cells in immune infiltration clusters. ^*^ *p* < 0.05; ^**^ *p* < 0.01; ^***^ *p* < 0.001; ns, not significant.

The abundance of 22 tumor-infiltrating immune cells in the TME of HCC was determined using the CIBERSORT algorithm, as depicted in **Supplementary Table S3**. In the immune microenvironment, M2 macrophages and resting memory CD4^+^ T cells were the most abundant (**Figure 2A**). The abundance of different tumor-infiltrating immune cells was found to be weakly to moderately correlated. Of the 22 immune cells, CD8^+^ T cells correlated best not only with immune score but also with CD4^+^ T cells (**Figure 2B**). In general, the correlation heatmap showed that T cell exhaustion was the main feature of immune cell dysfunction in the tumor microenvironment, which was one of the key links of tumor immunotherapy.

Considering the interindividual difference in the proportion of immune cell infiltration, unsupervised hierarchical clustering was performed on all samples. The optimal number of clusters was three (**Supplementary Table S4**), and t-SNE analysis confirmed that three subtypes of patients could be completely distinguished (**Figure 2C**). The clinical characteristic and immune cell proportions of each cluster are shown in **Figure 2D**. Cluster A exhibited the best prognosis, and cluster C possessed the worst outcome (**Figure 2E**). Furthermore, there were significant differences in the immune-checkpoint expressions (e.g., CTLA-4, PD-1, and TIM-3, **Figure 2F**) and HCC stage (**Supplementary Figures S2A, B**) among the three immune patterns, which were significantly correlated with tumor progression.

Then, the effect of distinct immune cell infiltration patterns on the clinical prognosis of HCC patients was further investigated. As shown in **Figure 2G**, the subjects in cluster A showed higher resting memory CD4^+^T cells, which were associated with favorable prognosis. Cluster B was defined by a high level of follicular helper T cell (Tfh) and M2 macrophages. Cluster C with a low level of CD8^+^ T cells and a high level of Treg cells had a pessimistic outcome. These results suggested that three clusters had distinct immune cell infiltration features in the composition.

### Identification of Gene Subtypes of HCC Based on Immune Profiles

**Figure 3:**
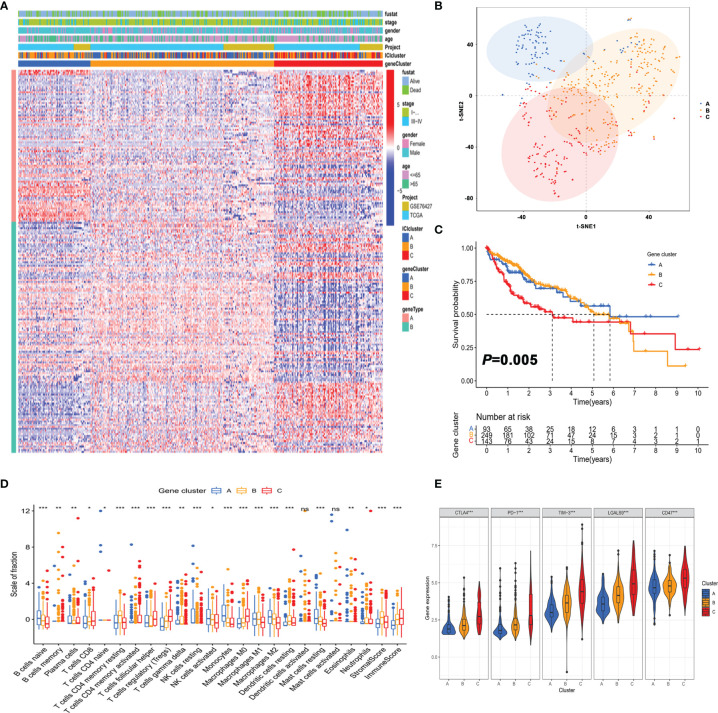
Identification of gene subtypes of HCC based on immune profiles. **(A)** Unsupervised clustering of differential gene expression among three immune infiltration clusters. **(B)** Confirmation of the clustering patterns using the t-SNE algorithm. **(C)** Kaplan–Meier curves for patients in the three clusters with log-rank *p* = 0.005. **(D)** Comparison of the proportion of tumor-infiltrating immune cells in three gene clusters. ^*^ *p* < 0.05; ^**^ *p* < 0.01; ^***^ *p* < 0.001; ns, not significant. **(E)** Comparison of the expression of immune checkpoints among distinct immune infiltration clusters. ^*^ *p* < 0.05; ^**^ *p* < 0.01; ^***^ *p* < 0.001.

To reveal the underlying biological features of distinct immunophenotypes, 214 genes were obtained by differential expression analysis (**Supplementary Table S5**). Three gene clusters were identified by the unsupervised hierarchical clustering, namely, gene subtypes A–C (**Supplementary Table S4**). The transcriptomic profiles of identified gene subtypes were depicted as a heatmap in **Figure 3A**, and t-SNE analysis confirmed that patients in three subtypes could be completely distinguished (**Figure 3B**). Through Kaplan–Meier plotter analysis, we found that patients in gene subtype A presented a favorable prognosis, while those in gene subtype C had a poor prognosis (**Figure 3C**).

As depicted in **Figure 3D**, the three gene clusters had distinct TME features. Expression levels of immune checkpoints such as PD-1 and CTLA4 were significantly different in the three gene subtypes (**Figure 3E**). Taken together, the concordance of immune profile-based genotypes with prognosis indicated that the molecular typing of HCC had important clinical prognostic significance.

### Construction and Functional Enrichment Analysis of the ICI Score Based on Immune Infiltration-Related Genes

**Figure 4:**
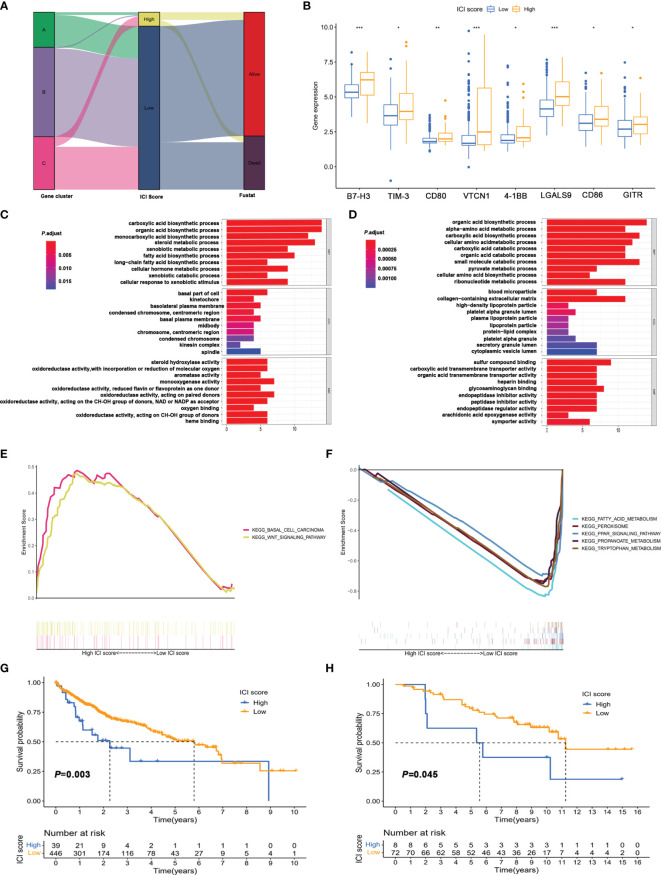
Construction of immune score and functional enrichment analysis based on immune infiltration-related genes. **(A)** Alluvial plot of distribution of the ICI score and prognosis in different immune infiltration gene clusters. **(B)** Expression of immune checkpoint in high and low ICI score groups. ^*^ *p* < 0.05; ^**^ *p* < 0.01; ^***^ *p* < 0.001; ns, not significant. **(C, D)** GO enrichment analysis of signature genes A **(C)** and B **(D)**. **(E, F)** Gene set enrichment analysis (GSEA) of high score **(E)** and low score group **(F)**. **(G)** Kaplan–Meier survival analysis of high score and low score groups divided based on the optimal cutoff value. **(H)** Kaplan–Meier survival analysis of high score and low score groups in the validation cohort (GSE10141).

To further obtain a quantitative indicator of immune cell infiltration in HCC patients, the Boruta algorithm was used to reduce the redundant genes to obtain the optimal feature genes (**Supplementary Table S5**) and PCA was performed to construct the ICI score for each patient. **Figure 4A** shows the ICI score distribution of HCC patients in three gene subtypes. The expression of genes associated with immune checkpoints was significantly increased in the high ICI score group (**Figure 4B**). Meanwhile, the ICI score was significantly positively correlated with ImmuneScore, but not StromalScore and ESTIMATEScore (**Supplementary Figure S3**), which implied that the ICI score established could better reflect the immune status of the TME.

**Figure 5:**
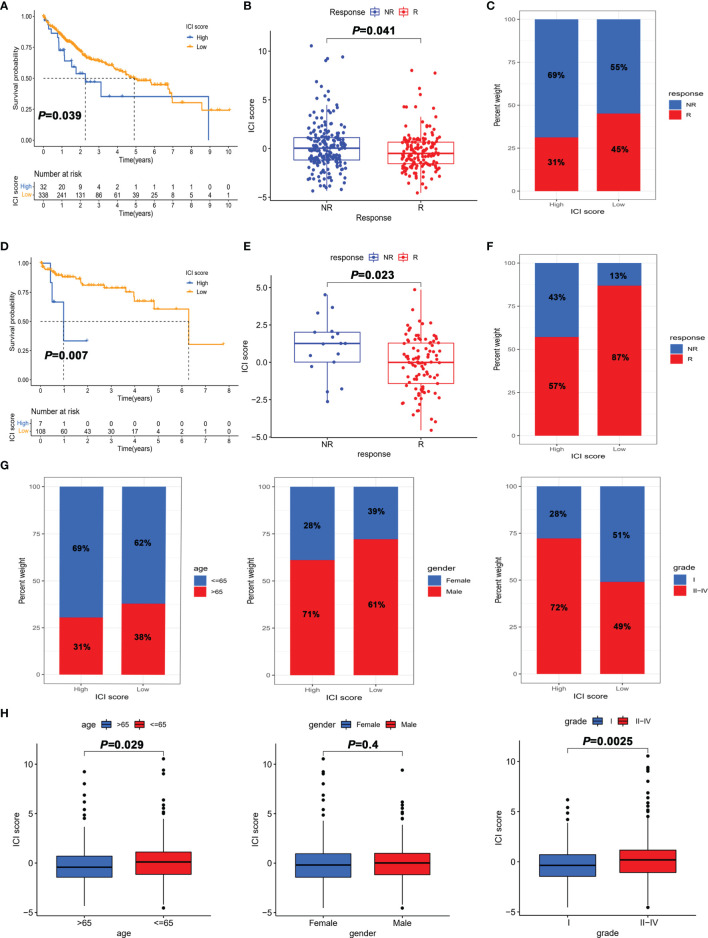
The role of the ICI score in the prediction of immunotherapeutic benefits. **(A)** Kaplan–Meier curves for patients with high and low ICI score in the TGCA-LIHC cohort. **(B)** Comparison of the ICI score in response and non-response to immunotherapy in the TGCA-LIHC cohort. **(C)** Percentage of objective response rate to immunotherapy for high score and low score groups in the TGCA-LIHC cohort. **(D)** Kaplan–Meier curves for patients with high and low ICI scores in the GSE76427 cohort. **(E)** Comparison of ICI scores in response and non-response to immunotherapy in the GSE76427 cohort. **(F)** Percentage of objective response rate to immunotherapy for the high score and low score groups in the GSE76427 cohort. **(G)** Percentage of clinical parameters for high score and low score groups. **(H)** Comparison of clinical parameters between high score and low score groups.

Additionally, the significant biological process of GO enrichment analysis for signature genes A and B is displayed in **Figures 4C, D**, respectively (**Supplementary Tables S8**, **S9**). The gene set enrichment analysis (GSEA) revealed that the differentially expressed genes for the high ICI score group were mainly involved in the Wnt/β-catenin pathway (**Figure 4E** and **Supplementary Table S10**). Metabolism pathways related to fatty acid, tryptophan, and propanoate were significantly enriched in the low ICI score group (**Figure 4F** and **Supplementary Table S11**). Kaplan–Meier plotter analysis revealed that the patients in the low ICI score group had poor prognosis than those in the high ICI score group (median OS 3.32 vs. 6.29 years, **Figure 4G** and **Supplementary Table S12**). In addition, the ICI score also exhibited good performance in predicting prognosis in external validation cohort (GSE10141, **Figure 4H**) and internal validation cohorts (TCGA-LIHC, **Figure 5A**; GSE76427, **Figure 5D**).

### The Role of the ICI Score in the Prediction of Immunotherapeutic Benefits

To further determine the role of the ICI score in predicting the benefit of immunotherapy, the ImmuCell analysis website was used to predict the therapeutic effect of immune checkpoint inhibitors based on the transcriptome. Patients with a high ICI score in both TCGA-LIHC and GSE76427 cohorts had better prognosis than those in the low score group (**Figures 5A, D** **)**. As shown in **Figures 5B, E**, the ICI score of the immunotherapy response group was lower than that of the non-response group. Besides, the objective response rate to immunotherapy was higher in the high ICI score group, which indicated that the ICI score was associated with objective response to immunotherapy (**Figures 5C, F** **)**. Overall, these data manifested that ICI score could better predict the response to immunotherapy.

To further explore the utility of the ICI score in predicting the clinic therapeutic benefits in HCC patients, we analyzed the correlation between ICI score and clinical parameters. As depicted in **Figures 5G, H**, patients aged >65 years and grade I showed a significantly lower ICI score than those aged < 65 years and grades II–IV. Notably, the proportion of early-stage HCC (grade I) in the low score group was 51%, which was much higher than the 28% in the high score group. Collectively, the results obtained could be important for optimizing current immunotherapies targeting the aging world populations and contribute to the development of precise treatment of early-stage HCC.

### The Correlation Between the ICI Score and Tumor Mutation Burden

**Figure 6:**
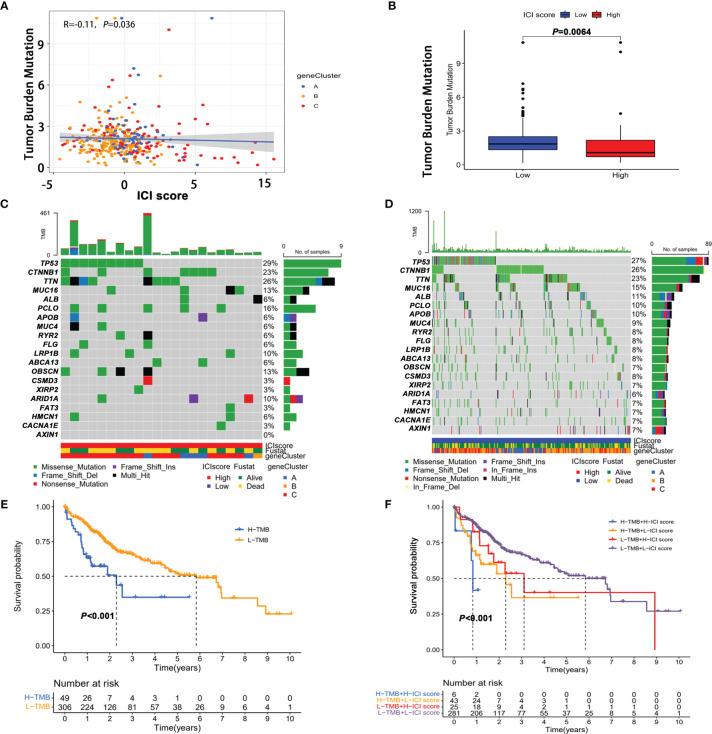
The correlation between the ICI score and tumor mutation burden (TMB). **(A)** Correlation between tumor mutation burden and ICI score. **(B)** Comparison of TMB in high score and low score groups. The oncoPrint plots of tumor-related gene mutations in the high ICI score **(C)** and low ICI score groups **(D)**. **(E)** Kaplan–Meier curves of overall survival in the high TMB and low TMB groups. **(F)** Kaplan–Meier survival curve for high/low TMB combined with high/low ICI score.

Considering that genetic instability was a hallmark of tumor and the significant clinical implications of TMB, we explored whether TMB status and ICI score play independent or synergistic roles in predicting prognosis. First, we sought to determine the TMB values (**Supplementary Table S13**) and compare the differences in TMB between patients with high and low scores. Correlation analysis showed that the ICI score was negatively correlated with TMB (**Figure 6A**). Moreover patients with a low ICI score showed a significantly higher TMB than those in the high ICI score group (**Figure 6B**). Then the distribution of the prevalent TOP 20 oncogenic drivers with the highest alteration frequency in the low and high ICI score groups is shown in **Figures 6C, D**. Among the 20 genes, the majority of genes were associated with increased TMB value except for ABCA13, APOB, ARID1A, AXIN1, and MUC4 (**Supplementary Figure S4**). After stratifying patients according to TMB status, ICI score subgroups still strongly correlated with patient prognosis regardless of TMB values (**Figures 6E, F** **)**, supporting the superior prognostic power of molecular features of the immune infiltration patterns.

### CTNNB1 Mutation Might Be Predictive of Response to Immunotherapy

**Figure 7:**
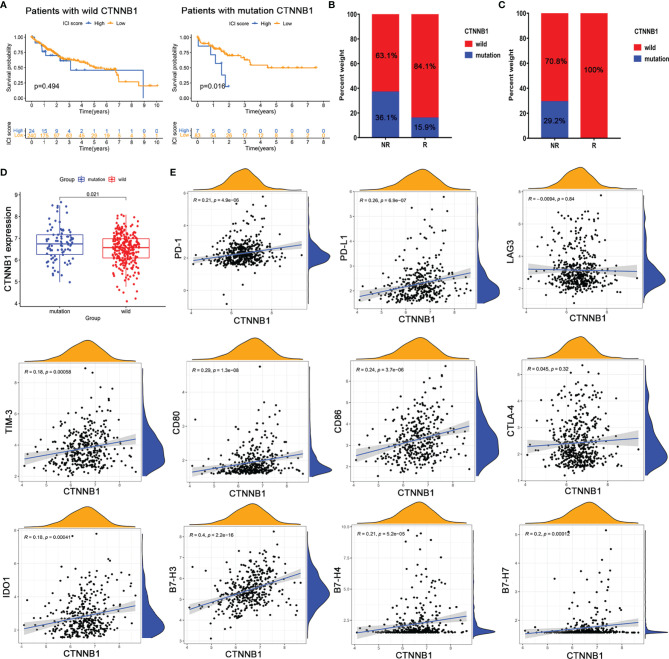
(CTNNB1 mutation might be predictive of response to immunotherapy. Kaplan–Meier survival curve of the **(A)** CTNNB1 wild-type group and **(B)** CTNNB1 mutation group in TCGA-LIHC. **(C)** CTNNB1 mutation status in the response and non-response groups from TCGA-LIHC (left) and cBioPortal database (right). **(D)** Comparison of the expression of CTNNB1 in wild-type and mutant groups. **(E)** Correlation between CTNNB1 expression and T-cell checkpoint expressions.

To further screen potential biomarkers for immunotherapy of HCC, we analyzed the top 20 oncogenic drivers with the highest alteration frequency. Among them, the significant difference in ICI score only existed between the patients with CTNNB1 mutation and those without CTNNB1 mutation (**Supplementary Figure S5**). Similarly, CTNNB1 status was also significantly related to the prognosis of patients with HCC (**Figure 7A**). Using the Kaplan–Meier curve analysis, we also found that only CTNNB1 expression was associated with poor outcomes (**Supplementary Figure S6**). Furthermore, in the TCGA-LIHC cohort, the mutation frequency of CTNNB1 was lower in the immunotherapy response group (15.9%) than in the non-response group (36.1%) (**Figure 7B**). A similar result was observed in the cBioPortal dataset, and none of the patients who responded to immunotherapy had CTNNB1 mutation (**Figure 7C**). The infiltration levels of T cells, B lineage, monocytic lineage, dendritic cells, neutrophils, and NK cells were significantly reduced in CTNNB1-mutated HCC patients (all *p* values were less than 0.05, **Supplementary Figure S7**). To sum up, CTNNB1 mutation tended toward higher TMB, higher ICI score, and lower immune cell infiltration, which better predicted immunotherapy response and may be a prognostic predictor in HCC patients.

To test whether CTNNB1 mutation affected CTNNB1 function, we compared the expression level of CTNNB1 wild type and mutant type and found that its expression was significantly upregulated in the CTNNB1-mutated group (**Figure 7D**). We then assessed the correlation between CTNNB1 expression and the expression of critical immune checkpoints that have emerged as biomarkers for the selection of HCC patients for immunotherapy. The results showed that CTNNB1 expression was weakly associated with the expression of classical T cell immune checkpoint pathway-related molecules (e.g., PD-1/PD-L1, LAG3/TIM-3, B7-1/B7-2/CTLA-4) (**Figure 7E**). Besides, there were no significant correlations between CTNNB1 expression and the majority of killer immunoglobulin-like receptors (e.g., KIR2DL1, KIR2DS4, and KIR3DL1) that play an essential role in the regulation of natural killer (NK) activity (**Supplementary Figure S8**). The findings partly explained why patients with CTNNB1 mutation were innate resistant to immune checkpoint inhibitors. Taken together, CTNNB1 mutation might serve as a predictive marker of response to immunotherapy, which contributed to the precise decision-making of immunotherapy in HCC patients.

## Discussion

Immunotherapy is emerging as a promising option for advanced HCC patients. A significant limitation of immunotherapy is that only a subset of HCC patients benefit from it. Given the individual heterogeneity of the immune environment, there is an urgent need to quantify the tumor immune infiltration patterns across individuals. In this study, we comprehensively analyzed the immune infiltration landscape of 491 HCC samples and categorized the HCC samples into three distinct immune infiltration subtypes. Furthermore, we established the ICI score to comprehensively quantify the immune environment of HCC, which might be an effective prognostic biomarker and predictor for evaluating immunotherapy response.

Currently, the mechanism of immunotherapy is based on the activation of T cells, especially CD8^+^ T, because of their role in tumor cell cytolysis. In the study, we found that CD8^+^ T cells had the best correlation with other 21 immune cells. It indicated that CD8^+^ T cells were the main cause of immune exhaustion in the immune microenvironment, which was consistent with the core concept of clinical immunotherapy. Besides, CD8^+^ T cells were also highly related to CD4^+^ T cells, which might partly explain the insufficient immune response of CD8^+^ T cells to eliminate tumors and why scientists had shifted their focus to the “supporting role” of CD4^+^ T cells. Overall, the results further confirmed that T cells were the core cells of immunotherapy, which partially explained why immune checkpoints targeting T cells could generate markedly specific immune responses.

Growing evidence manifests that immune cell dysfunction in the TME promoted immune suppression, thereby promoting tumorigenesis and progression (29–31). For instance, CD4^+^ T cells, CD8^+^ T cells, and Treg cells play important roles in tumor recurrence, metastasis, and immunotherapy response (32, 33). Here, we found that cluster A with the best prognosis was characterized by the highest CD4^+^T cell infiltration and exhibited an active immune phenotype, which was routinely so-called hot tumor (34–36). The cluster C with the worst prognosis had a low CD8^+^/regulatory T cell ratio, which implied an immune-cold phenotype (37, 38). However, the results of the ESTIMATE algorithm showed no significant difference in the ImmuneScore of the three clusters, which seemed to be inconsistent with the CIBERSORT algorithm. It implied that immune components, rather than the quantity, were more likely to affect the prognosis of immune subtypes.

Based on the differential genes among distinct immune infiltration patterns, we established the ICI score to fully quantify immune infiltration patterns by PCA analysis. The high ICI score group exhibited poor prognosis, and the expressions of immune checkpoints such as PD-L1 and TIM-3 were significantly increased. The prognostic value of the ICI score was validated in the external validation cohort (GSE10141). Age is associated with many changes in immune function, primarily a reduction in the number of lymphocytes, especially T cells, and a decrease in the diversity of the T-cell receptor repertoire (39, 40). Stratified analysis showed that patients aged >65 years and grade I showed significantly lower ICI score than those aged <65 years and grades II–IV, suggesting that the ICI score was important for optimizing current immunotherapy targeting an aging world population and developing precise treatments for early HCC. Through GSEA, we found that genes involved in the high ICI score group were enriched in the Wnt/β-catenin pathway, now commonly defined as immune exclusion HCC class, also known as HCC “cold” tumors (41). Furthermore, the ImmuCell analysis website predicted response to immunotherapy in the TCGA-LIHC and GSE76427 cohorts. The results showed a lower ICI score in the immunotherapy response group. In contrast, the objective response rate of immunotherapy was lower in the high ICI score group. Taken together, these data manifested that the ICI score could better predict the response to immunotherapy and had important clinical significance.

Previous studies have shown that TMB is a predictive biomarker of the efficacy of immunotherapy, and the higher the TMB, the better the efficacy of immunotherapy (42, 43). In the study, our current study showed that high TMB was a poor prognosis factor for patients with HCC, which was consistent with HCC being a genetic disease requiring only four mutations on average. Besides, we also found a significant difference in the mutation frequency of multiple genes between the high and low ICI score groups. The ICI score was significantly negatively correlated with TMB, with a correlation coefficient of -0.17, and TMB was significantly increased in patients with a low ICI score. The stratified analysis showed that the ICI score could synergistically predict HCC patients’ prognosis with TMB, which implies that the molecular alterations may interfere with cell-to-cell communication between infiltrating immune cells during tumorigenesis.

Considering the limitation of the ICI score that can only be obtained by transcriptome analysis of surgical specimens, we analyzed the somatic mutation genes in the high and low score groups to screen potential biomarkers for HCC immunotherapy and found that the ICI score was significantly increased in patients with CTNNB1 mutation. Previous studies have shown that 30% of HCC patients harbor CTNNB1 mutation, which may represent those with innate resistance to immune checkpoint inhibitors (41–44). Compared with wild-type CTNNB1, HCC patients with CTNNB1 mutation in TCGA-LIHC and cBioPortal databases were resistant to immunotherapies, suggesting that CTNNB1 might represent the biomarkers for predicting resistance to immune checkpoint inhibitors. Moreover, T cells, B lineage, monocytic lineage, dendritic cells, neutrophils, and NK cells exhibited lower infiltration levels in CTNNB1-mutated HCC patients. This was in line with previous studies showing that CTNNB1 mutation was characterized by an immune excluded class, so-called cold tumors (41). The reason is that, on the one hand, CTNNB1 mutation caused its expression to be significantly upregulated, which was closely related to the activation of the Wnt/β-catenin pathway. On the other hand, CTNNB1 expression was weakly correlated with immune checkpoint expression, which can partially explain why patients with CTNNB1 mutation were refractory to antibodies targeting PD-1/PD-L, or LAG3, or KIR (45). However, CTNNB1 expression was moderately correlated with B7-H3 (CD276), a new checkpoint target for cancer immunotherapy with an expression frequency of 91.8% in HCC (46), which suggested that CTNNB1-mutated HCC patients might escape immunotherapy through the non-classical immune checkpoint pathway, providing a novel direction for the treatment of CTNNB1-mutated HCC patients and precise decision-making for HCC management. Certainly, all the results were based on theoretical analysis of transcriptome data. The effective target and molecular mechanism for the treatment of CTNNB1-mutated HCC patients still need to be further explored and verified.

## Conclusion

In summary, we performed a comprehensive analysis of the immune landscape in the TME of HCC. We found that the difference in immune infiltration patterns of the immune microenvironment of HCC was closely related to tumor heterogeneity, treatment response, and prognosis. Therefore, the systematic evaluation of the tumor immune infiltration model in this study could help determine the optimal immunotherapy strategy for patients, which had important clinical significance.

## Data Availability Statement

The datasets presented in this study can be found in online repositories. The names of the repository/repositories and accession number(s) can be found in the article/**Supplementary Material**.

## Author Contributions

XZ and QK designed and directed the completion of the project. XT was involved in writing of the review and editing. YG performed the data analysis and drawing. XT and KR performed the data download and clinical data collection. YG and HG performed the statistical analyses. JY supervised the study. All authors contributed to the article and approved the submitted version.

## Funding

This work was supported by the National Key Research and Development Program of China (No. 2020YFC2008304) and the Medical Science and Technology Joint Project of Henan Province (No. LHGJ20210282).

## Conflict of Interest

The authors declare that the research was conducted in the absence of any commercial or financial relationships that could be construed as a potential conflict of interest.

## Publisher’s Note

All claims expressed in this article are solely those of the authors and do not necessarily represent those of their affiliated organizations, or those of the publisher, the editors and the reviewers. Any product that may be evaluated in this article, or claim that may be made by its manufacturer, is not guaranteed or endorsed by the publisher.

## Supplementary Material

Supplementary Table S1 Detailed description of the clinical characteristics for HCC samples from TCGA-LIHC, GSE76427 and GSE10141.

Supplementary Table S2 ImmuneScore, StromalScore and ESTIMATEScore quantified for each HCC sample by ESTIMATE algorithm.

Supplementary Table S3 The abundance of 22 types of tumor‐infiltrating immune cells using the CIBERSORT method.

Supplementary Table S4 Unsupervised clustering for all HCC patients according to the proportion of immune cells.

Supplementary Table S5 Unsupervised clustering of differentially expressed genes among three immune infiltration clusters.

Supplementary Table S6 Differentially gene expression profiles of the three immune infiltration clusters.

Supplementary Table S7 Signature gene expression profiles for different immunity patterns based on Boruta algorithm.

Supplementary Table S8 The gene ontology analysis of ICI signature genes A ranked according to the adjusted P-value.

Supplementary Table S9 The gene ontology analysis of ICI signature genes B ranked according to the adjusted P-value.

Supplementary Table S10 KEGG pathway enrichment analysis of high ICI Score group.

Supplementary Table S11 KEGG pathway enrichment analysis of low ICI Score group.

Supplementary Table S12 ICI score of HCC patients from TCGA-LIHC and GSE76427.

Supplementary Table S13 Mutation status of HCC samples from TCGA-LIHC (0, absence of mutations; 1, presence of mutations).

Supplementary Table S14 Mutation status of HCC samples from TCGA-LIHC (0, absence of mutations; 1, presence of mutations).

Supplementary Figure S1 The workflow of the study design.

Supplementary Figure S2 Comparison of stages of HCC among distinct immune infiltration clusters. (A) HCC stage; (B) The proportion of HCC stage divided into early (I) and late HCC (II-IV).

Supplementary Figure S3 Correlations between ImmuneScore/StromalScore/ESTIMATEScore and ICI score.

Supplementary Figure S4 Correlations between TMB value and expressions of the TOP 20 oncogenic drivers with the highest alteration frequency.

Supplementary Figure S5 Comparison of ICI score between HCC samples with mutation and without mutation of the TOP 20 oncogenic drivers with the highest alteration frequency.

Supplementary Figure S6 Effect of expressions of the TOP 20 oncogenic drivers with the highest alteration frequency on prognosis.

Supplementary Figure S7 Comparison of the infiltration levels of immune cells between HCC samples with and without CTNNB1 mutation.

Supplementary Figure S8 Correlations between CTNNB1 expression and expression of killer immunoglobulin-like receptors.

## Abbreviations

HCC, hepatocellular carcinoma; TMB, tumor mutational burden; ICI, immune cell infiltration; TME, tumor microenvironment; t-SNE, t-distributed stochastic neighbor embedding; GSEA, gene set enrichment analysis; PCA, principal component analysis.
