# Supplementary figures and images for: The Heterogeneity of Immune Cell Infiltration Landscape and Its Immunotherapeutic Implications in Hepatocellular Carcinoma
Source: Front Immunol. 2022 Mar 10;13:861525. doi: 10.3389/fimmu.2022.861525 (PMC8959995; doi:10.3389/fimmu.2022.861525)

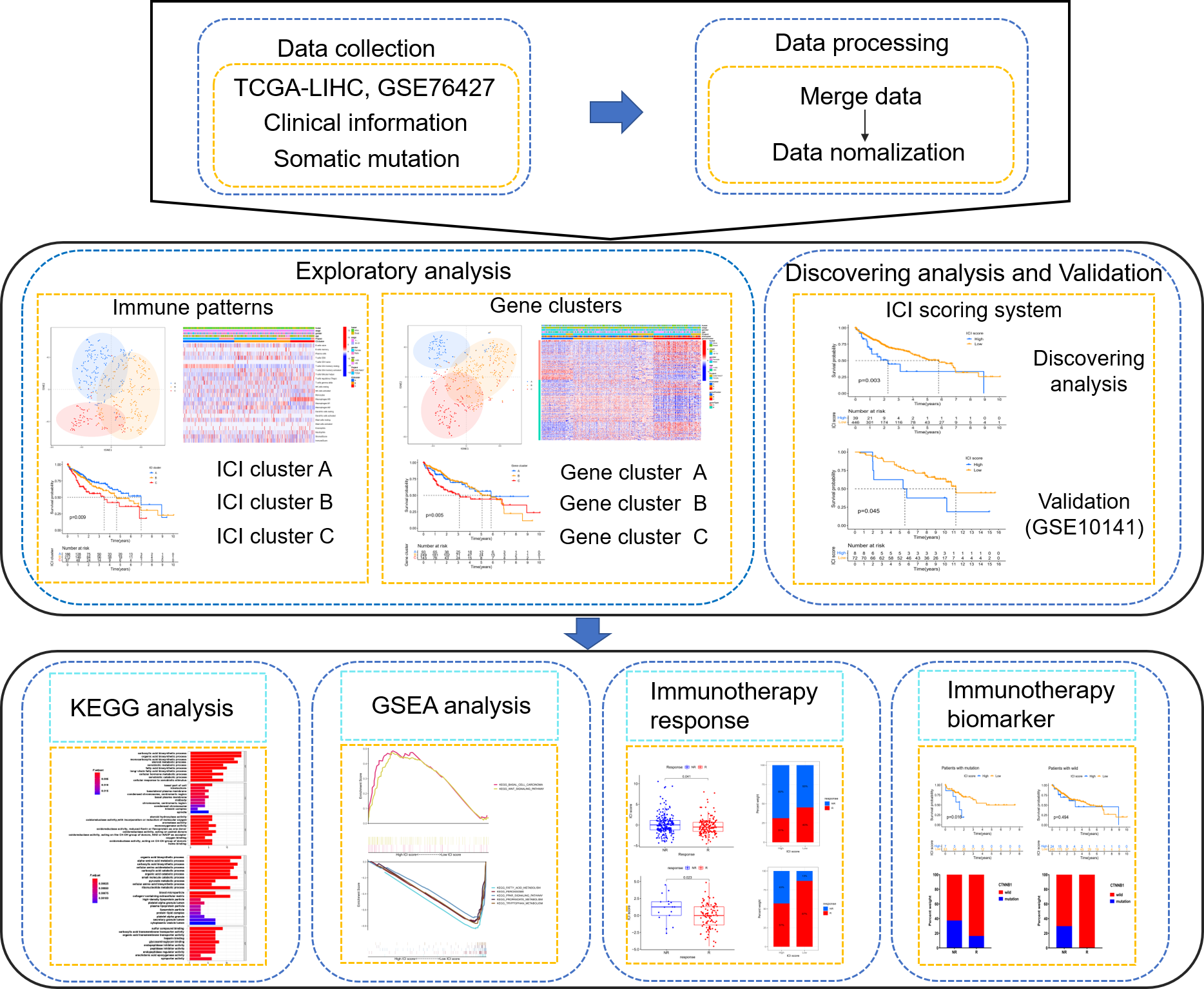

Supplement: Supplementary Figure S1 — The workflow of the study design. [file Image_1.tif]

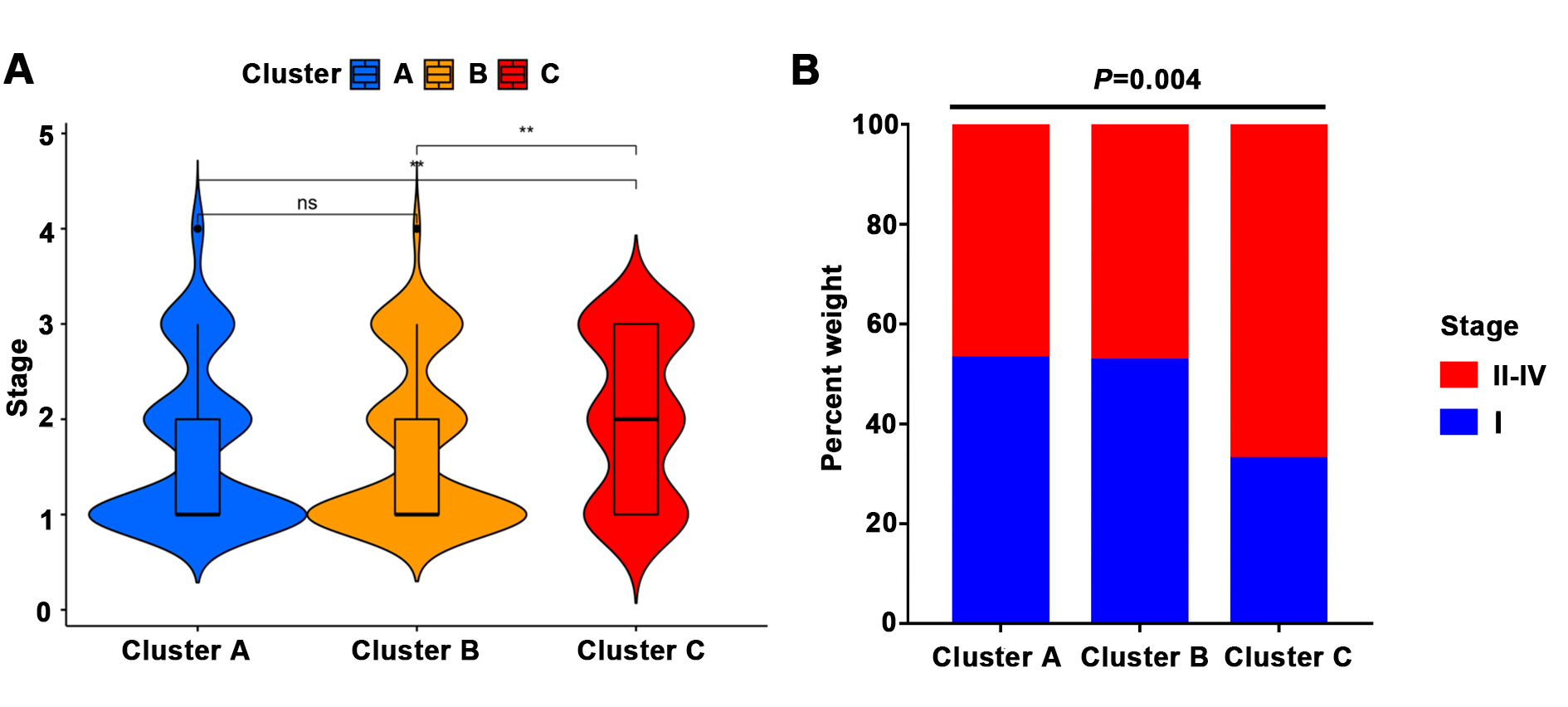

Supplement: Supplementary Figure S2 — Comparison of stages of HCC among distinct immune infiltration clusters. (A) HCC stage; (B) The proportion of HCC stage divided into early (I) and late HCC (II-IV). [file Image_2.tif]

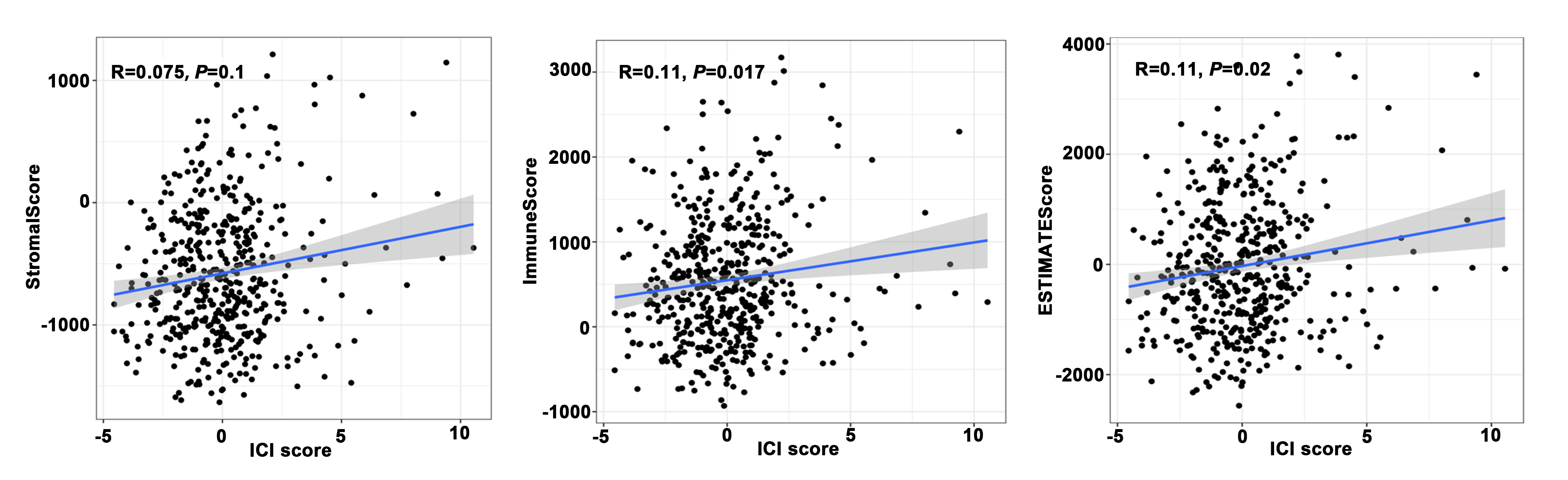

Supplement: Supplementary Figure S3 — Correlations between ImmuneScore/StromalScore/ESTIMATEScore and ICI score. [file Image_3.tif]

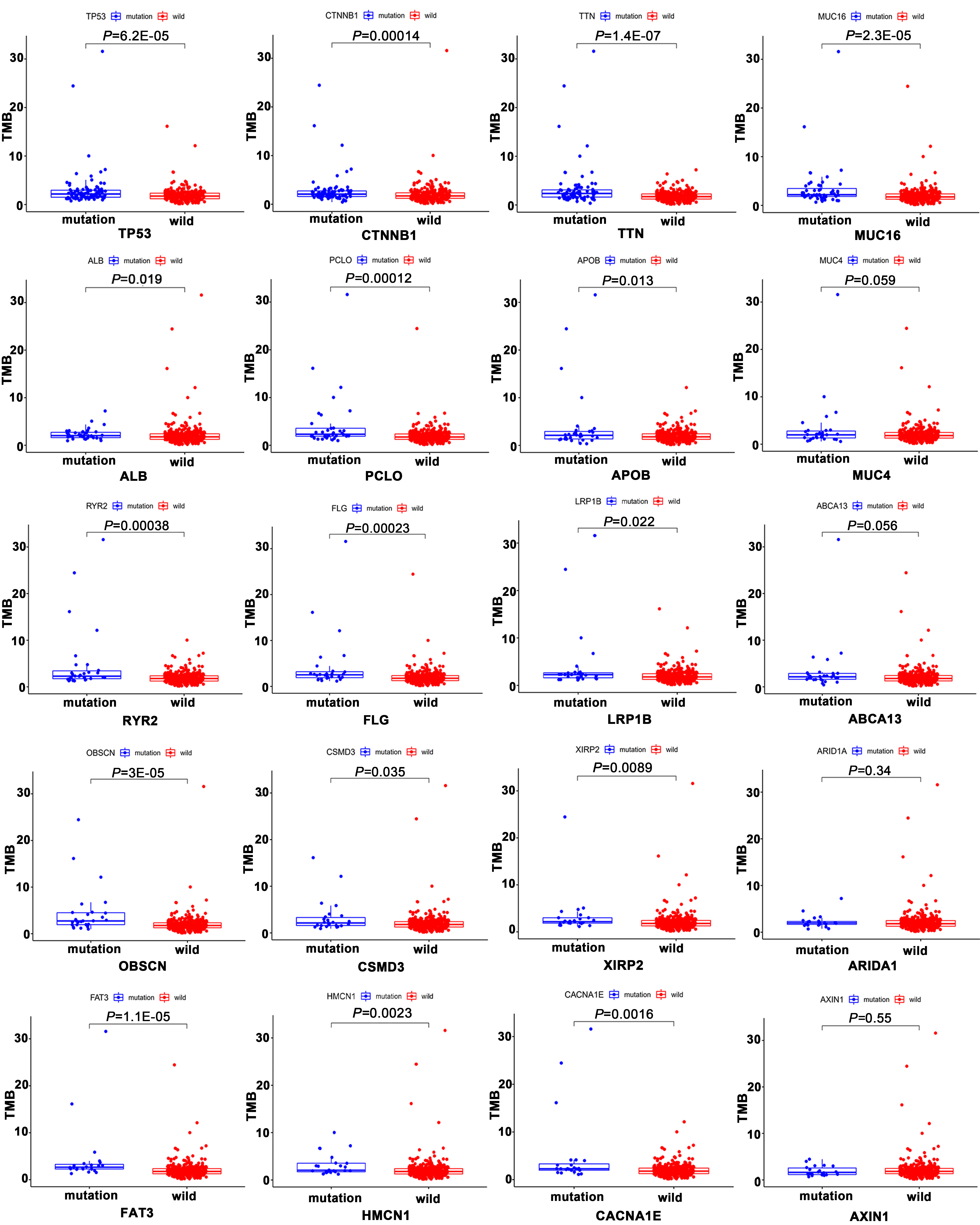

Supplement: Supplementary Figure S4 — Correlations between TMB value and expressions of the TOP 20 oncogenic drivers with the highest alteration frequency. [file Image_4.tif]

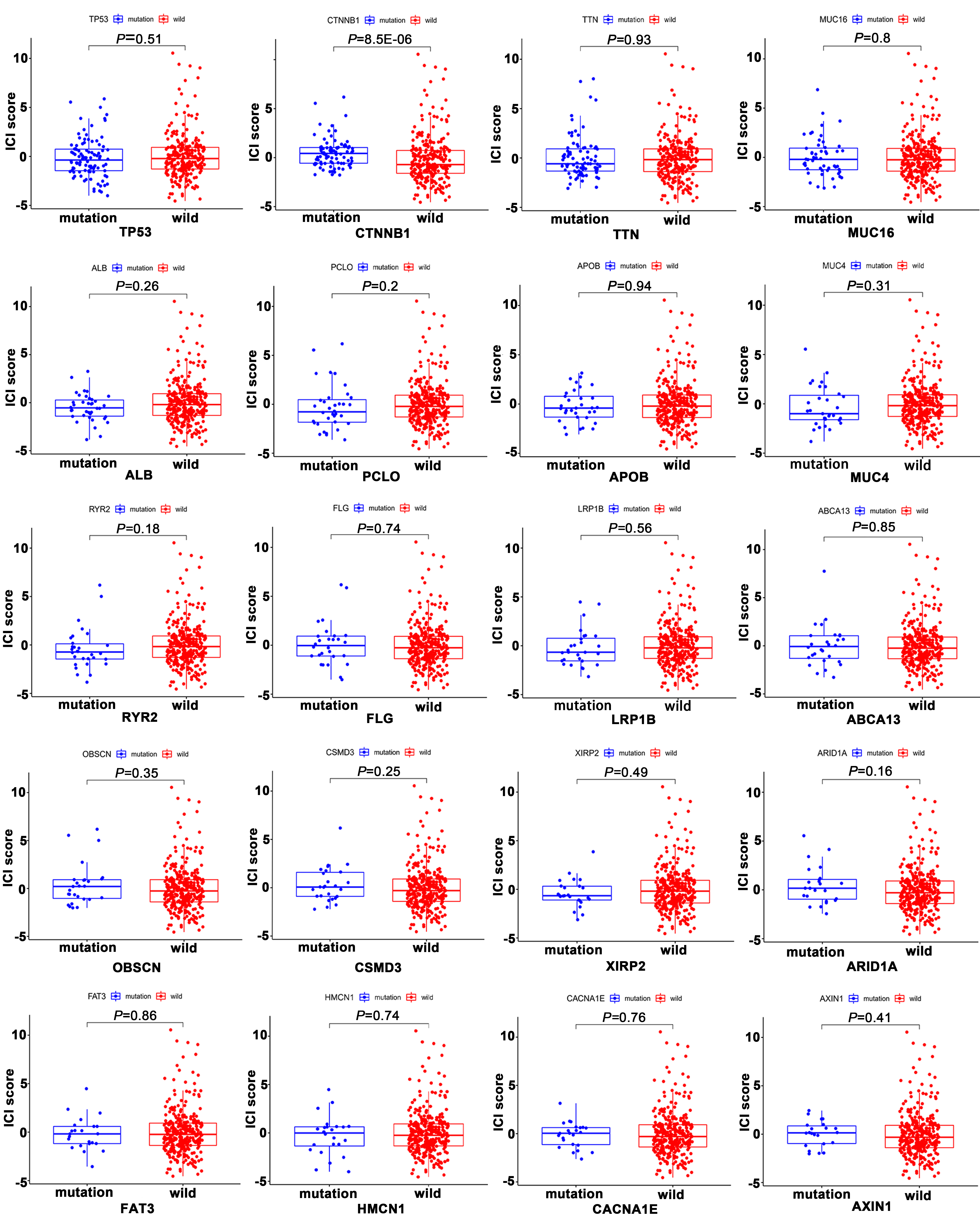

Supplement: Supplementary Figure S5 — Comparison of ICI score between HCC samples with mutation and without mutation of the TOP 20 oncogenic drivers with the highest alteration frequency. [file Image_5.tif]

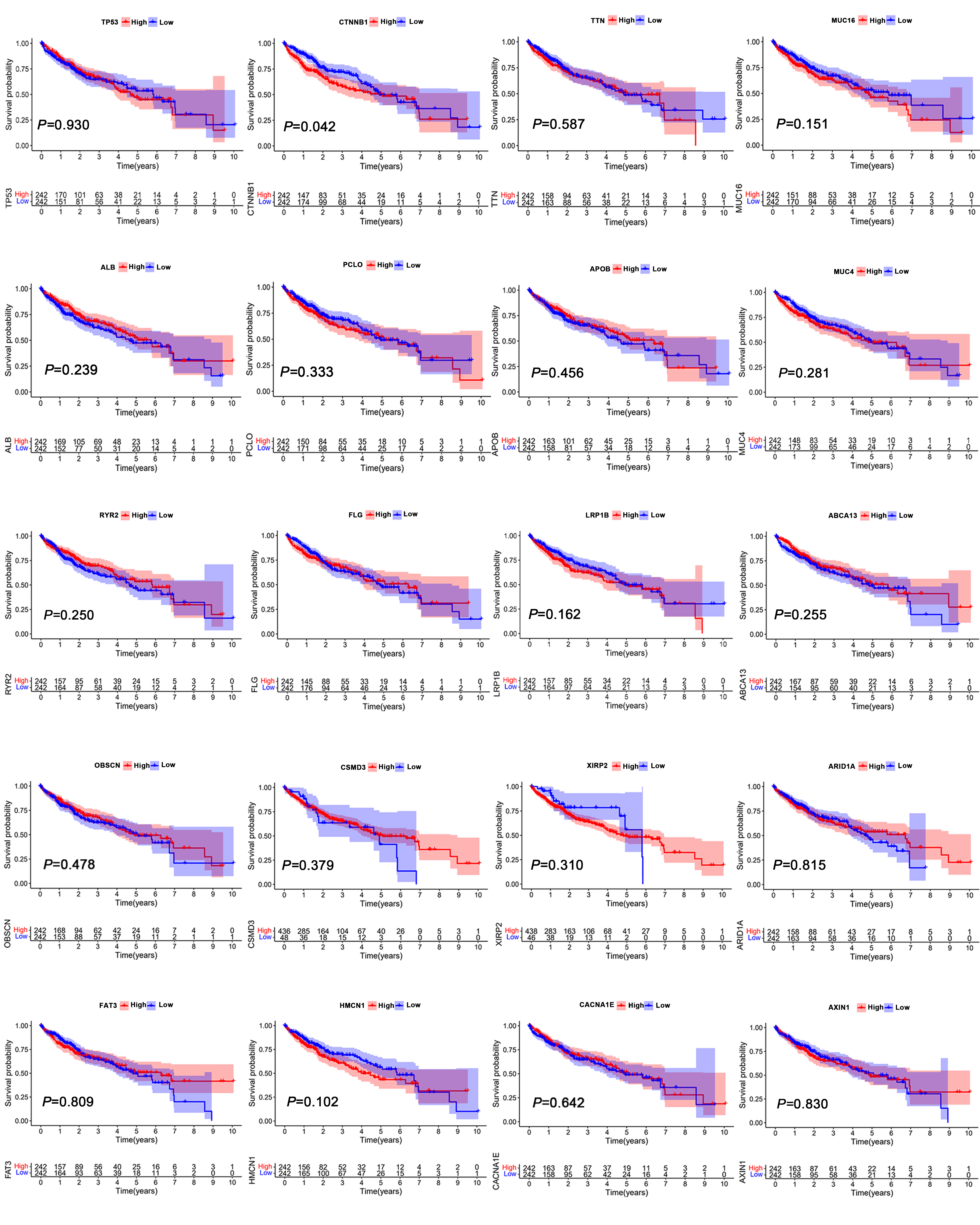

Supplement: Supplementary Figure S6 — Effect of expressions of the TOP 20 oncogenic drivers with the highest alteration frequency on prognosis. [file Image_6.tif]

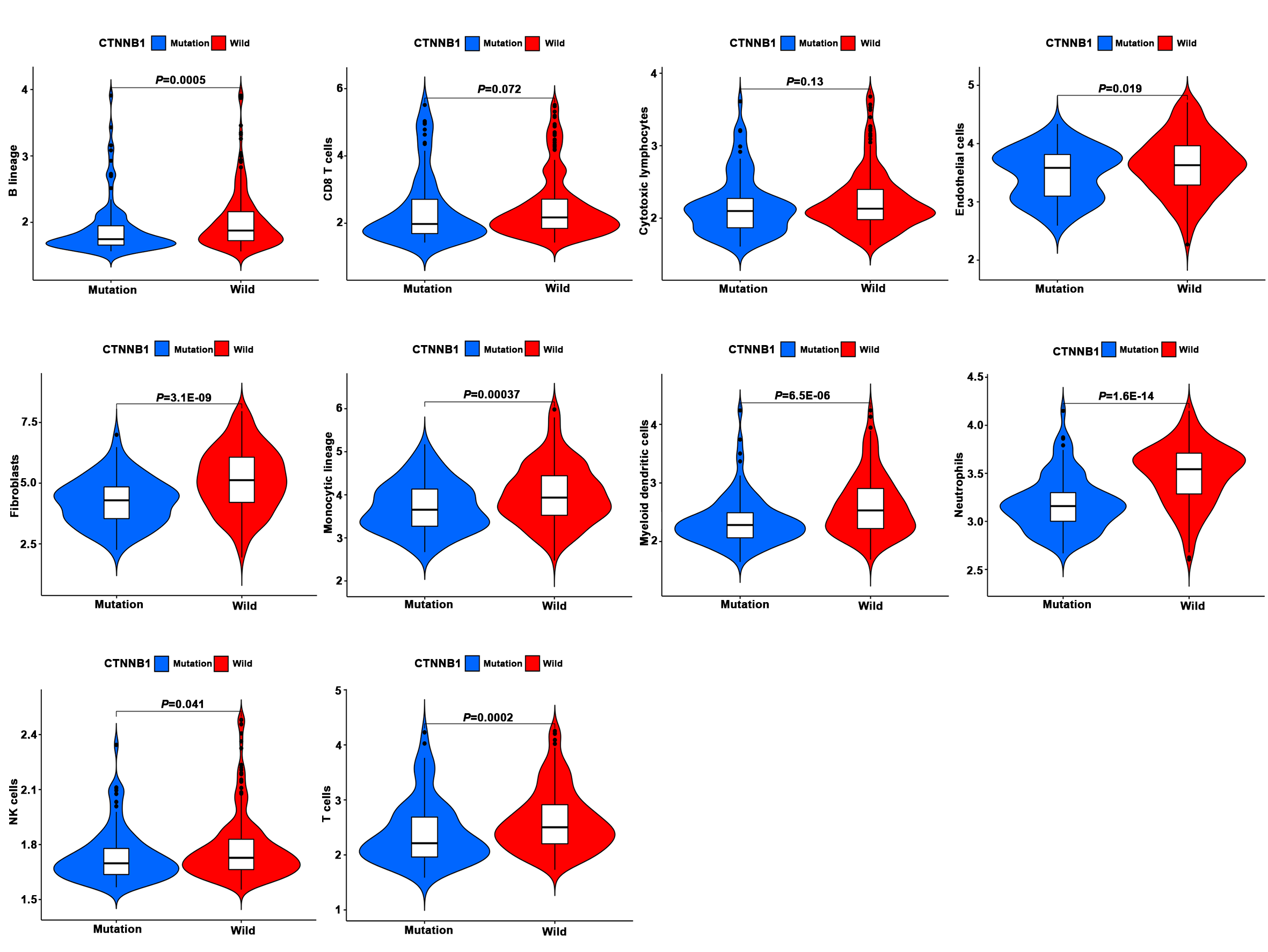

Supplement: Supplementary Figure S7 — Comparison of the infiltration levels of immune cells between HCC samples with and without CTNNB1 mutation. [file Image_7.tif]

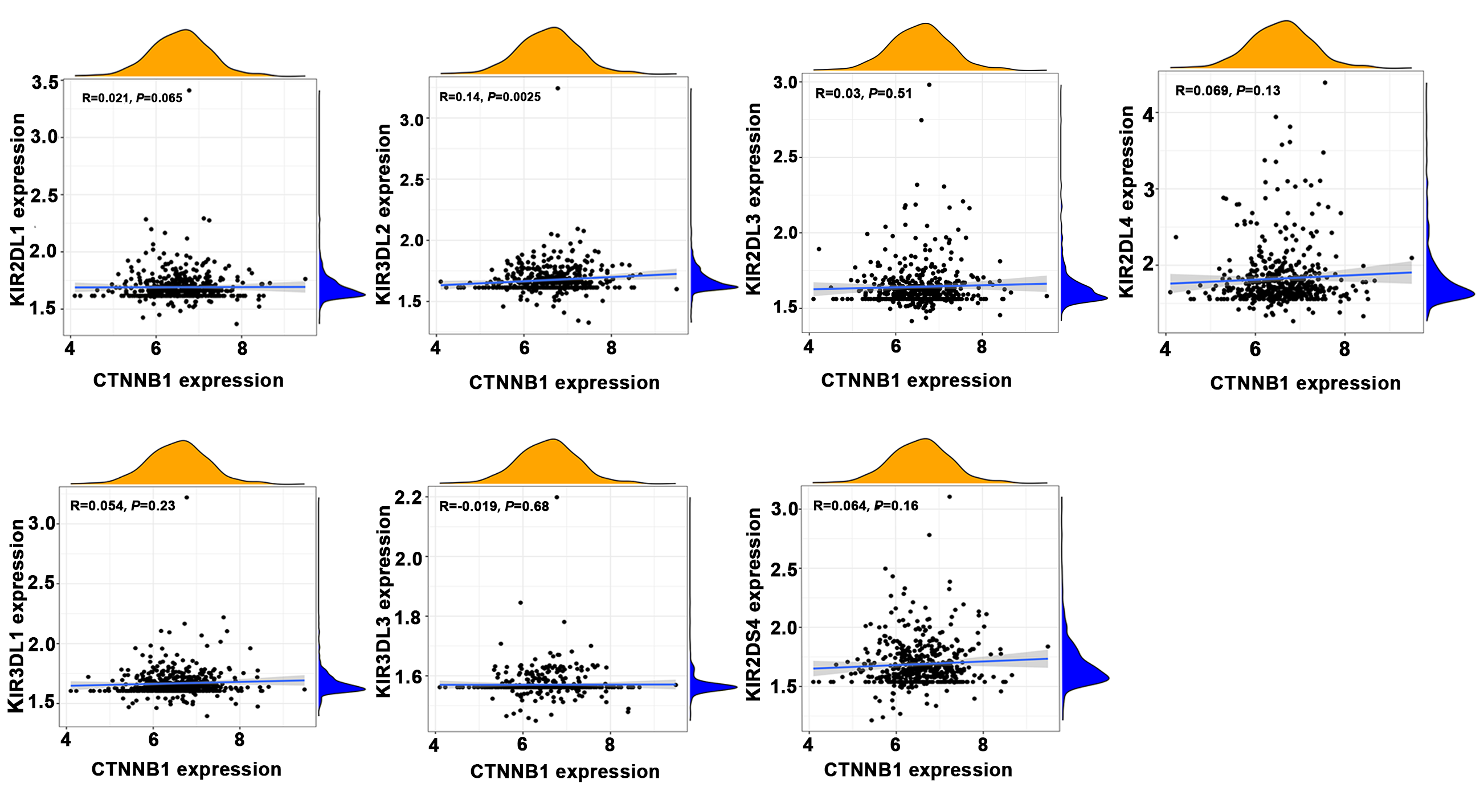

Supplement: Supplementary Figure S8 — Correlations between CTNNB1 expression and expression of killer immunoglobulin-like receptors. [file Image_8.tif]
